# Associations between incremental exercise capacity and multimodal brain characteristics across training levels

**DOI:** 10.3389/fnhum.2026.1753838

**Published:** 2026-07-31

**Authors:** Keying Zhang, Ling Jiang, Yuyan Wang, Qing Yan, Chunmei Cao, Dong Zhang

**Affiliations:** 1 Department of Physical Education, Southeast University, Nanjing, China; 2 Institute of Artificial Intelligence in Sports, Capital University of Physical Education and Sports, Beijing, China; 3 Division of Sports Science and Physical Education, Tsinghua University, Beijing, China

**Keywords:** aerobic fitness, brain plasticity, DC, fALFF, GMV, resting-state fMRI

## Abstract

**Background:**

Previous work has shown that endurance training is associated with structural and functional plasticity in prefrontal, hippocampal, cerebellar and network level regions using both cross sectional and longitudinal designs. The present study extends this foundation by examining how key endurance indicators relate to multimodal neural characteristics across different training levels.

**Methods:**

Thirty-two male participants were classified into high level endurance athletes, moderately trained runners and healthy active controls. All participants completed structural MRI and resting state functional MRI together with graded exercise testing to assess maximal oxygen consumption, relative maximal oxygen consumption, lactate threshold and individual lactate threshold (ILT). Gray matter volume, fractional amplitude of low frequency fluctuations and degree centrality were extracted from predefined regions of interest. Correlation analyses were first conducted in the full sample and within subgroups, followed by false discovery rate correction within imaging modalities. Associations surviving FDR correction were further evaluated using Spearman rank correlations, bootstrap confidence intervals for subgroup findings, and covariate-adjusted partial correlations as sensitivity analyses.

**Results:**

Across all participants, incremental exercise-capacity indicators were associated with multimodal neural characteristics in prefrontal, premotor, precuneus, temporal, and posterior cerebellar regions, with additional negative associations observed in the thalamus and inferior cerebellum in the uncorrected analyses. After FDR correction, the surviving associations became more selective. Subgroup analyses suggested descriptively different association patterns across training levels, but these subgroup findings were less stable in sensitivity analyses and should therefore be interpreted cautiously.

**Conclusion:**

Incremental exercise-capacity indicators were associated with distributed multimodal brain characteristics, and several core associations remained robust after multiple-comparison correction and supplementary analyses. The subgroup results suggested stage-related differences in brain–physiology coupling, particularly a broader pattern in moderately trained individuals and a more focal prefrontal-associated profile in high-level athletes, although these findings still require cautious interpretation.

## 1 Introduction

Endurance performance has traditionally been interpreted through the lens of peripheral physiological limitations such as cardiovascular capacity, muscular metabolism and oxygen delivery. However, emerging evidence from exercise neuroscience demonstrates that endurance is fundamentally constrained and regulated by central neural processes (Calderón-Montero et al., 2021; Hettinga et al., 2017). Contemporary theoretical frameworks, including the central governor model (Venhorst et al., 2018), the psychobiological model of endurance and the neural efficiency hypothesis (Tornero-Aguilera et al., 2022), converge on the notion that the brain plays a decisive role in regulating motor output, allocating attentional and motivational resources, monitoring internal states and maintaining systemic homeostasis during prolonged effort. Neuroimaging studies have consistently identified the involvement of the prefrontal cortex, premotor areas, parietal regions, the cerebellum and large-scale networks such as the default mode and frontoparietal control networks during sustained physical exertion (Port et al., 2024; Raichlen et al., 2016; Reid et al., 2016; Wu et al., 2024). These regions support critical functions including effort perception, motor planning, error monitoring and adaptive control under fatigue. Such evidence suggests that endurance capacity reflects not only peripheral physiological potential but also a complex, distributed and centrally regulated neural process. Yet despite accumulating evidence of exercise-related brain plasticity, it remains unclear how individual differences in endurance capacity are systematically represented within the brain.

More and more neuroimaging studies have shown that endurance training is associated with structural and functional adaptations across multiple brain regions, including increases in gray matter volume (GMV) within sensorimotor areas, alterations in default mode and executive networks, and enhanced cerebellar-cortical integration (Cassady et al., 2018; Geisler et al., 2024; Raichlen et al., 2016; Yan et al., 2025; Zhang et al., 2024). These findings highlight that endurance training induces distributed neural plasticity rather than isolated regional changes. However, much of the existing literature relies on binary comparisons between athletes and non-athletes, which overlook the continuous nature of endurance development and the possibility that neural adaptations may differ across training stages. Furthermore, prior work has typically examined single imaging modalities in isolation and therefore provides a fragmented view of how structural and functional features collectively support endurance performance. Our recent cross-sectional and longitudinal studies extended this literature by demonstrating that endurance training may follow a stage-dependent trajectory of neural adaptation, characterized by broader and more heterogeneous plasticity at moderate training levels and more focused changes at higher levels of specialization (Zhang et al., 2025; Zhang et al., 2024). Yet these studies did not address a fundamental question: whether individual differences in endurance capacity, indexed by maximal oxygen consumption and lactate thresholds, are systematically reflected in multimodal neural features, and whether the relationship between endurance capacity and brain organization varies across different stages of training.

Theoretical advances in neurophysiology and exercise metabolism increasingly emphasize that endurance performance emerges from tight coupling between neural regulation and metabolic efficiency. Models such as neurovascular coupling (DiNuzzo et al., 2024; Sundqvist et al., 2022), the lactate shuttle hypothesis (Wu et al., 2023; Zhu et al., 2025) and integrative theories of metabolic control (Furlan and Petrus, 2023; Shoemaker and Gros, 2024) propose that sustained exercise relies on coordinated adaptations that support efficient energy delivery, resource allocation and cognitive-motor integration. Exercise-related metabolic adaptation may also involve oxidative stress signaling, inflammatory modulation, and glial responses, which may provide additional pathways through which training intensity shapes neural plasticity (Radak et al., 2013; Damay et al., 2023; Eo and Leem, 2023; Palasz et al., 2025). These perspectives imply that brain plasticity related to endurance is likely to manifest across multiple neural dimensions rather than within a single structural or functional domain. Multimodal MRI provides a powerful framework for capturing these complementary aspects of neural organization. GMV reflects long-term structural capacity and training-related morphological remodeling (Ashburner and Friston, 2000). Fractional amplitude of low-frequency fluctuations (fALFF) indexes intrinsic regional neural activity, which may relate to baseline readiness or regulatory efficiency (Zou et al., 2008). Degree centrality (DC) quantifies network-level integration and accessibility, capturing how effectively a region interacts with distributed circuits (Burdette et al., 2010). Together, these metrics allow characterization of structural reserves, local functional dynamics and large-scale network coordination within a unified neural system supporting endurance. Similarly, physiological indicators such as maximal oxygen consumption and lactate thresholds serve as robust and quantifiable proxies of endurance capacity (Staff et al., 2023; van der Zwaard et al., 2021). VO₂max reflects upper limits of oxygen utilization and cardiorespiratory function, whereas lactate thresholds index metabolic stability and sustainable exercise capacity (Staff et al., 2023). Both metrics align closely with central regulatory demands described by neural-metabolic coupling theories. Despite their scientific importance, little is known about how these endurance measures map onto multimodal brain features or whether such brain-endurance relationships differ across individuals with distinct training experiences.

Taken together, the present study aimed (1) to examine the associations between incremental exercise-capacity indicators, including maximal oxygen consumption, lactate threshold, and ILT, and multimodal brain characteristics, and (2) to explore whether individuals with high, moderate, and no training experience show descriptively different patterns of brain-physiology coupling. We hypothesized that: (1) incremental exercise-capacity indicators would be positively associated with neural features in regions related to motor regulation, such as the prefrontal cortex, premotor cortex, parietal regions, and the cerebellum; and (2) individuals with different training levels might show different descriptive patterns of brain-physiology coupling. In this way, the study extends our previous training-level framework by examining how continuous physiological indicators relate to multimodal brain characteristics across different training backgrounds.

## 2 Materials and methods

### 2.1 Study design

This study was an observational, cross-sectional, exploratory, correlational study. Brain structure and resting-state fMRI were obtained simultaneously in the same sample, along with endurance capacity, to test statistical associations between the two.

**Figure 1:**
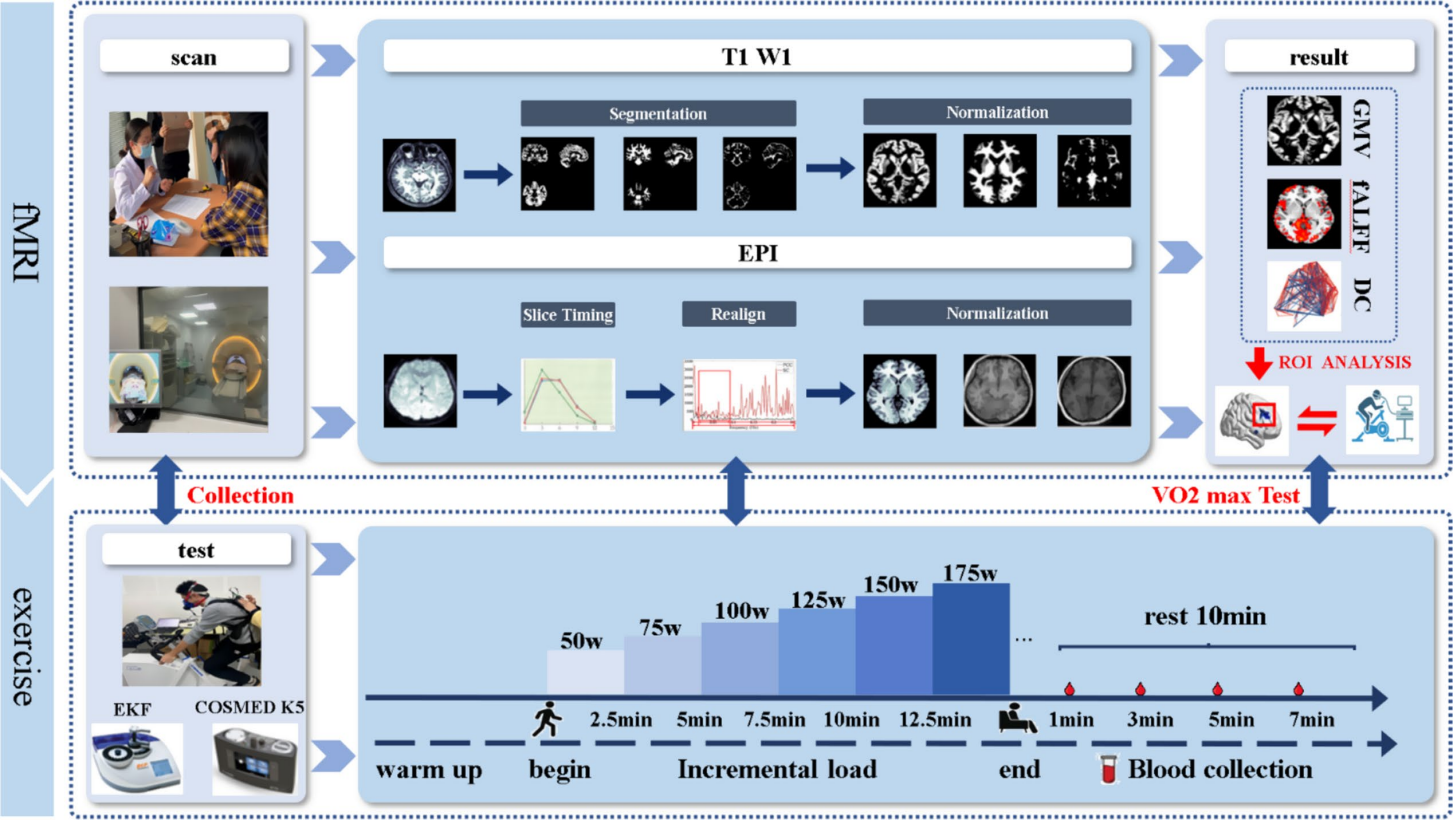
An overview of the experimental design. MRI comprised two sequences: T1-weighted structural imaging to quantify structural plasticity (GMV) and functional plasticity (fALFF and DC). During the graded incremental load test, gas exchange variables, heart rate, and earlobe capillary blood lactate were recorded.

All participants first underwent MRI scanning and then completed a graded exercise test (GXT). To standardize physiological status, participants were instructed to avoid vigorous exercise for 48 h before the graded test; to abstain from food, exercise, alcohol, and coffee within 3 h prior to testing; and to rest on the test day, avoiding any clearly strenuous physical activity. Before the test, participants provided written informed consent and completed a Physical Activity Readiness Questionnaire (PAR-Q) (Craig et al., 2003; Lee et al., 2011). The schematic overview of the procedure is shown in Figure 1.

### 2.2 Participants

This study initially enrolled 35 male participants, including 10 high-level middle- and long-distance runners, 10 moderate-level long-distance runners, and 15 healthy university controls. Inclusion criteria were male sex, age 18–30 years, good health condition, right-handedness, and no history of musculoskeletal disorders, chronic disease, long-term medication use, or other major illnesses. Exclusion criteria included standard MRI contraindications (e.g., metal implants, claustrophobia, pacemaker) and recent injury or surgery. After safety screening for MRI, 3 high-level athletes were excluded due to contraindications (recent musculoskeletal injury or metallic tattoos incompatible with MRI), yielding a final analytic sample of 32 participants, including: (1) The high endurance capacity group (HG) comprised seven high-level middle- and long-distance runners (18–25 years) with 4–8 years of specialized endurance training, typically 5–7 sessions per week of approximately 2.5 h each. (2) The moderate endurance capacity group (MG) comprised 10 long-distance runners (18–28 years) who engaged in regular but non-specialized endurance training (e.g., sports club or team-based training) for 1–4 years, averaging about three sessions per week for 1–2 h per session. (3) The control group (CON) comprised 15 healthy male university students (19–26 years) who were physically active but had no history of systematic endurance training. The basic demographic data of the three subject groups are presented in Table 1.

**Table 1:** Basic demographic data of subjects.

Variable	HG (*n* = 7)	MG (*n* = 10)	CON (*n* = 15)	*F*	*p*
Age (year)	21.6 ± 2.4	23.6 ± 3.3	21.7 ± 2.3	1.838	0.177
Height (cm)	177.6 ± 3.6	173.5 ± 4.9	175.3 ± 5.5	1.372	0.270
Weight (Kg)	66.4 ± 4.6	67 ± 5.2	65.9 ± 7.1	0.092	0.912
BMI (Kg/cm^2^)	21.04 ± 0.85	22.26 ± 1.58	21.42 ± 1.78	1.424	0.257
Endurance training period (year)	6.1 ± 1.6	2.6 ± 1.5	0^**##^	75.246	<0.001

**Indicates a highly significant difference compared to the high-level group (*p* < 0.01), and ^##^indicates a highly significant difference compared to the moderate-level group (*p* < 0.01).

### 2.3 Image data acquisition and processing

Brain imaging data were acquired using a 3.0 T Philips Achieva scanner with a 32-channel phased-array head coil. To investigate both structural and functional neuroplasticity, two types of images were collected: high-resolution T1-weighted anatomical scans and single-shot gradient-echo planar imaging (EPI) sequence.

For structural analysis, T1-weighted images were obtained using a standardized sequence with the following parameters: flip angle = 8°, 180 contiguous slices, slice thickness = 1 mm, acquisition matrix = 232 × 230, and field of view = 230 × 230 mm. The T1 images were processed using voxel-based morphometry (VBM) implemented in the VBM8 toolbox. The processing pipeline included skull stripping, spatial normalization using the DARTEL algorithm, tissue segmentation into gray matter, white matter, and cerebrospinal fluid, and spatial smoothing with an 8 mm isotropic Gaussian kernel. This procedure yielded whole-brain GMV maps for subsequent analysis.

For functional imaging, blood-oxygen-level-dependent (BOLD) signals were acquired using an EPI sequence. The imaging parameters were: TR = 2,000 ms, TE = 30 ms, flip angle = 90°, 37 axial slices, slice thickness = 3 mm (with a 0.5 mm slice gap), matrix size = 80 × 80, FOV = 230 × 230 mm, and voxel size = 2.87 × 2.87 × 3.50 mm^3^. During the resting-state scan, participants were instructed to keep their eyes closed, remain awake, and let their thoughts pass without focusing on anything. To minimize scanner noise interference, participants wore earplugs and noise-reducing headphones. In addition, head motion was restrained using foam padding around the head. Functional data preprocessing included removal of the first five volumes to allow for magnetic field stabilization, slice timing correction, head motion realignment, co-registration to structural images, spatial smoothing with an 8 mm FWHM Gaussian kernel, and nuisance regression (including white matter and cerebrospinal fluid signals, as well as 24 motion parameters). After preprocessing, whole-brain fALFF and DC maps were computed.

Region-of-interest (ROI) analyses were then conducted on the processed fALFF, DC, and GMV maps. Spherical ROIs (6 mm radius) were defined according to peak coordinates reported in previous endurance-training neuroimaging studies 9, 14. After merging overlapping or anatomically adjacent clusters, a total of 23 unique ROIs were identified across the three imaging indices. These ROIs encompassed key regions within the frontal cortex (prefrontal cortex, medial frontal cortex, dorsolateral prefrontal cortex, medial superior frontal gyrus, premotor cortex), limbic system (hippocampus, parahippocampal gyrus, entorhinal cortex, amygdala), parietal–occipital cortices (precuneus, superior parietal lobule, angular gyrus, lateral occipital cortex), temporal regions (inferior temporal gyrus, temporal lobe/pole), basal ganglia (putamen/striatum), as well as the insula, thalamus, and multiple cerebellar subregions (posterior lobules, apex, vermis). Mean parameter estimates within each ROI were extracted using the WFU PickAtlas toolbox for subsequent statistical analyses.

### 2.4 Graded exercise testing and endurance capacity assessment

Endurance capacity was evaluated using a GXT to obtain absolute maximal oxygen consumption (VO₂max), relative VO₂max, lactate threshold intensity (LT), and individual LT (Beltz et al., 2016).

Cycle ergometer and protocol. The GXT was performed on a Monark LC4 cycle ergometer (Perez-Suarez et al., 2018), which uses a mechanical resistance mechanism system and supports constant power and resistance modes with pointer, mechanical, and electronic calibration. Seat height was adjusted to the level of the greater trochanter, a heart-rate strap and a metabolic mask were fitted, and pedal straps were secured. The formal test procedure consisted of 5 min of rest, incremental load cycling, and 10 min of recovery. Workload started at 50 W and increased by 25 W every 2.5 min until termination criteria were met, including any of the following: fulfillment of VO₂max criteria, rating of perceived exertion (RPE) ≥ 19, symptoms such as palpitations, chest discomfort, dyspnea, nausea, pallor, or headache, or failure of heart rate to rise with increasing workload accompanied by inability to continue.

Gas exchange and VO₂max determine. An Italy COSMED K5 portable metabolic system was used to record gas exchange data during the GXT. VO₂max was deemed achieved when at least two criteria were present: a plateau or decrease in VO₂ despite increasing workload, respiratory exchange ratio ≥ 1.10, heart rate ≥ 180 beats per minute, or RPE ≥ 19. If no clear plateau occurred and the participant reached volitional exhaustion, the highest VO₂ attained was taken as VO₂max. Relative VO₂max was calculated by dividing VO₂max by body mass in kilograms.

Heart rate and lactate measurements. Heart rate monitoring and sampling schedule. Heart rate was continuously monitored with a Firstbeat device. Before testing, resting heart rate, resting blood lactate concentration, and resting oxygen consumption were recorded. During GXT, earlobe capillary blood was sampled in the last 20 s of each workload stage, with heart rate and RPE recorded simultaneously. During recovery after test cessation, heart rate was recorded at the end of each minute up to minute 10, and additional earlobe blood samples were collected at the end of minutes 1, 3, 5, and 7. Lactate sampling and analysis. Earlobe capillary blood was obtained using a sterile safety lancet. After wiping away, the first drop, blood was collected with a glass capillary tube and transferred to an anticoagulant-treated sample tube and mixed thoroughly. Blood lactate concentration was measured using a benchtop glucose–lactate analyzer (EKF C_line Clinic, EKF, Germany). The analyzer was calibrated with standard solutions before each batch of measurements. Each sample was analyzed in duplicate and the mean value was reported. If the two readings differed by more than 5 percent, the sample was reanalyzed. The lactate threshold was defined as the lowest power output at which blood lactate concentration exceeded the resting value by more than 1.0 mmol·L^−1^and continued to rise thereafter. The ILT was determined using the Dmax method, by fitting the power-lactate curve and identifying the point with the greatest perpendicular distance from the straight line connecting the resting and peak points. Threshold intensity was expressed as absolute power in watts and as relative power, calculated as a percentage of the power corresponding to VO₂max.

All physiological indices were derived from cycle ergometry and therefore primarily reflect tolerance to graded maximal effort and central/metabolic responses, rather than sport-specific endurance performance.

### 2.5 Statistical analysis

Statistical analyses were conducted by SPSS 25.0. To examine the relationships between brain plasticity and endurance capacity, Pearson correlations were computed between signal values from each ROI and the following endurance indices: absolute VO₂max, relative VO₂max, lactate threshold, and ILT. All variables were verified to meet normality assumptions. To control for the potential inflation of Type I error due to multiple comparisons, false discovery rate (FDR) correction was applied using the Benjamini–Hochberg procedure. FDR correction was conducted within predefined families of tests, separately for each imaging modality (VBM, fALFF, and DC) across all ROIs and physiological variables.

Associations surviving FDR correction were further evaluated using nonparametric Spearman rank-correlation analyses as sensitivity analyses. For subgroup findings, bootstrap 95% confidence intervals (5,000 resamples) were computed to evaluate the stability of correlation estimates given the modest sample sizes. In addition, covariate-adjusted partial correlation analyses were performed for key FDR-surviving associations to assess the influence of major available confounders. For GMV-based findings, age and intracranial volume (ICV) were included as covariates; for the other imaging–physiology associations, major available demographic and physiological confounders were adjusted where appropriate. Statistical results are reported as correlation coefficients (*r*) with corresponding *p* values, and partial correlation coefficients where applicable. FDR-corrected *q* values are additionally reported when relevant. Results surviving FDR correction (*q* < 0.05) were considered statistically significant, whereas uncorrected findings (*p* < 0.05) were interpreted as exploratory.

## 3 Results

### 3.1 Data quality and test completion

All participants successfully completed the MRI scanning procedures, and all neuroimaging data met the quality-control criteria for further analysis. All participants also completed the GXT and reached at least two termination criteria required for determining VO₂max. Lactate-related indicators were obtained for most of the sample; however, two participants did not yield valid lactate measurements due to insufficient peripheral blood volume at sampling, resulting in missing values for lactate threshold and ILT.

### 3.2 GXT performance and aerobic capacity

**Figure 2:**
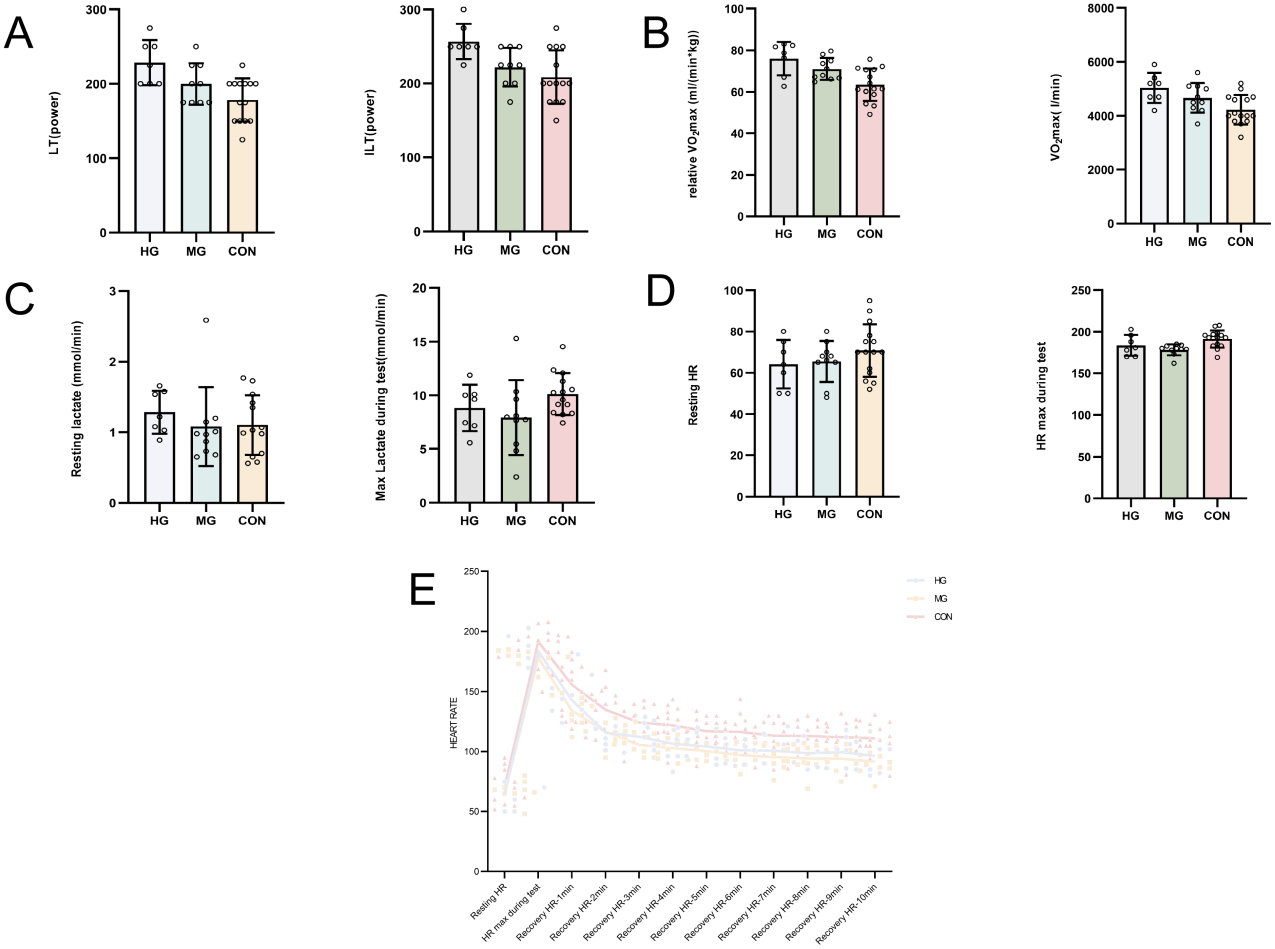
Results of GXT performance and aerobic capacity. **(A)** Group comparisons and associations of VO₂-related indicators, showing differences in maximal oxygen uptake and related aerobic capacity parameters. **(B)** Comparisons of LT (power)–related indicators, illustrating group differences in endurance thresholds and load-dependent performance. **(C)** Group differences in resting lactate and maximal lactate levels during the test, reflecting baseline metabolic status and exercise-induced lactate responses. **(D)** Comparisons of heart rate–related indicators (resting HR and maximal HR), demonstrating cardiovascular responses across groups. **(E)** Heart rate trajectories during the GXT and subsequent recovery period, showing the dynamic patterns of exercise-induced HR elevation and recovery among the three groups.

Workload performance. During the GXT, participants demonstrated clear group differences in workload attainment. The high-endurance group (*n* = 7) reached levels 9–12 (250–325 W; 282.1 ± 27.8 W), the moderate-endurance group (*n* = 10) reached levels 8–11 (225–300 W; 260.0 ± 24.2 W), and the control group (*n* = 15) reached levels 7–11 (200–300 W; 242.0 ± 32.7 W). The maximal workload achieved differed significantly among the three groups (*F* = 4.60, *p* = 0.018; Figure 2B).

VO₂max and relative VO₂max. Significant group differences were observed for VO₂max (*F* = 5.655, *p* = 0.008) and relative VO₂max (*F* = 8.153, *p* = 0.002). *Post hoc* analyses showed that the high-endurance group had significantly higher VO₂max than the control group (*p* = 0.003), and significantly higher relative VO₂max than both the control group (*p* = 0.001) and the moderate-endurance group (*p* = 0.015; Figure 2A).

Lactate threshold indicators. No significant differences were found in resting lactate concentration (*F* = 0.478, *p* = 0.625) or peak lactate concentration reached during the test (*F* = 2.123, *p* = 0.139; Figure 2C). However, significant group differences were observed in lactate threshold (*F* = 6.975, *p* = 0.004) and ILT (*F* = 5.667, *p* = 0.009). The high-endurance group showed a significantly higher lactate threshold than the control group (*p* = 0.001), and significantly higher ILT than both the control group (*p* = 0.002) and the moderate-endurance group (*p* = 0.034; Figure 2B).

Heart rate measures. Significant differences were found in maximal heart rate (*F* = 5.517, *p* = 0.009), with the high-endurance group exhibiting a significantly higher value than the control group (*p* = 0.003). No significant differences were observed in resting heart rate (*F* = 1.051, *p* = 0.362) or percentage of age-predicted maximal heart rate reached (*F* = 3.238, *p* = 0.054; Figure 2D). Although overall recovery trends were similar across groups, heart rate during minutes 1–10 of the recovery phase showed significant between-group differences (all *p* < 0.01; Figure 2E).

In summary, the three groups exhibited significant differences in workload performance, VO₂max, lactate threshold measures, and maximal heart rate, with the high-endurance group consistently demonstrating the highest levels across these indicators. No significant group differences were observed for resting lactate, peak lactate concentration, resting heart rate, or the percentage of age-predicted maximal heart rate achieved.

### 3.3 Associations between neural plasticity and endurance performance

**Figure 3:**
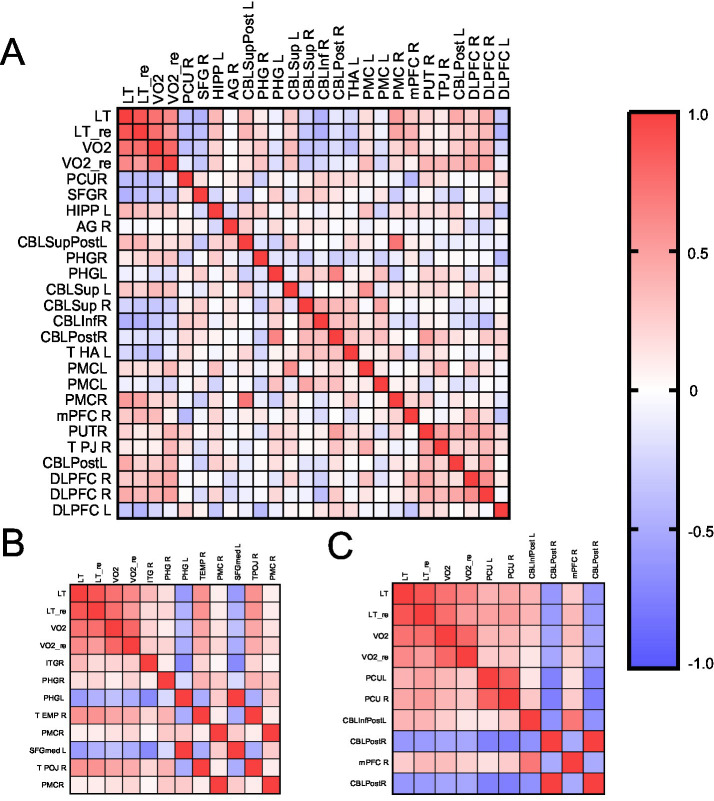
Correlation matrix among multimodal neuroimaging features and physical performance indicators. Panels **(A–C)** present correlation heatmaps illustrating the associations between four aerobic performance indicators (VO₂, VO₂_re, LT, LT_re) and three neuroimaging metrics: **(A)** Voxel-Based Morphometry (VBM), **(B)** Degree Centrality (DC), and **(C)** fractional Amplitude of Low-Frequency Fluctuations (fALFF). All values are standardized correlation coefficients. FDR-surviving associations are emphasized in the main text, and key findings were further evaluated using Spearman rank correlations, covariate-adjusted sensitivity analyses, and bootstrap confidence intervals where applicable.

In the uncorrected analyses, multiple nominal associations were observed between endurance-related physiological measures and multimodal neural indices across frontal, parietal, temporal and cerebellar regions (see Figure 3). These uncorrected findings suggested a distributed but exploratory pattern of associations with structural and functional features distributed across key frontal, parietal, temporal and cerebellar regions (see Figure 3). Maximal oxygen consumption was positively associated with GMV in the right dorsolateral prefrontal cortex (*r* = 0.392, *p* = 0.024) and medial frontal cortex (*r* = 0.345, *p* = 0.049), as well as with fALFF in the bilateral precuneus (left *r* = 0.375; right *r* = 0.378, both *p* < 0.05). DC in the right temporal region and the fronto-parieto-temporal junction showed among the strongest associations (both *r* = 0.459, *p* = 0.007), suggesting a distributed but exploratory pattern of associations. Relative VO₂max demonstrated a similar pattern, with the strongest link seen in the right dorsolateral prefrontal cortex (*r* = 0.497, *p* = 0.003) (see Figure 3A).

Lactate threshold exhibited comparatively stronger nominal relationships, particularly with the right premotor cortex (*r* = 0.522, *p* = 0.003), the right dorsolateral prefrontal cortex (*r* = 0.438, *p* = 0.014), and DC in temporal and fronto-parieto-temporal regions (both *r* = 0.569, *p* = 0.001). ILT closely mirrored this pattern, with prominent associations in the precuneus (right *r* = 0.513, *p* = 0.003) and fronto-parieto-temporal junction (*r* = 0.561, *p* = 0.001). Negative associations emerged primarily in the thalamus and inferior cerebellum, such as the cerebellar GMV correlating negatively with ILT (*r* = −0.502, *p* = 0.004), suggesting possible involvement of load-sensitive mechanisms, although these effects should be interpreted cautiously given the multiple testing burden.

**Figure 4:**
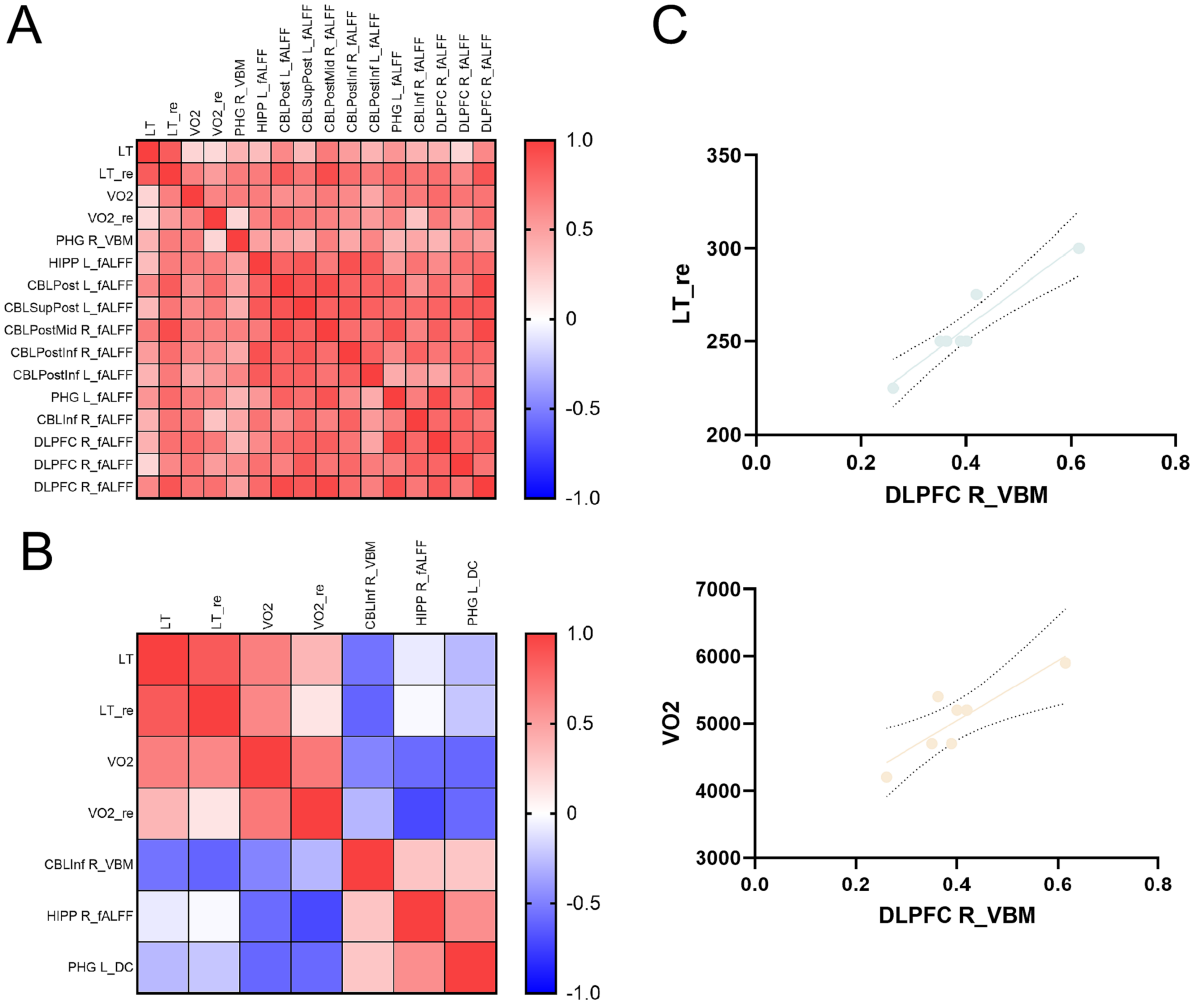
Key brain regions associated with performance variables across the three participant groups **(A–C)**. Panels **(A–C)** show the key brain–performance correlations for the three participant groups: **(A)** MG group, **(B)** CON group, and **(C)** HG group. Each part presents the associations between aerobic performance indicators (VO₂, VO₂_re, LT, LT_re) and the corresponding group-specific neuroimaging metrics. All values reflect standardized correlation coefficients. Notably, in the CON group **(B)**, significant correlations are observed only for VO₂ and LT_re. Group-specific associations are shown for descriptive purposes; formal interaction testing across groups was not performed, and subgroup findings should be interpreted cautiously given the sample sizes.

Meanwhile, after controlling for multiple comparisons using the false discovery rate (FDR) procedure and conducting nonparametric Spearman sensitivity analyses, the pattern of associations became more selective. Across modalities, the surviving effects converged on a limited set of regions. For VBM, the LT-related associations surviving FDR correction were located in the right premotor cortex, right inferior cerebellum, and right medial superior frontal region. For fALFF, the surviving LT-related associations involved the bilateral precuneus, left inferior posterior cerebellum, right posterior cerebellum, and right medial frontal region. For DC, significant effects were confined to the right temporal region and the right fronto-parieto-temporal junction across LT, LT_re, VO2, and relative VO2. Nonparametric Spearman analyses further supported the robustness of the key VBM findings, the bilateral precuneus and right posterior cerebellum in the fALFF analyses, and the LT/LT_re-related DC effects, whereas several other associations were attenuated. Together, these results indicate that the neural correlates of graded maximal-effort tolerance are more focal andselective than suggested by the uncorrected analyses (Figure 4).

To further assess the influence of potential confounding factors, covariate-adjusted partial correlation analyses were performed for the key associations surviving FDR correction. For GMV-based findings, age and intracranial volume were included as covariates; for the other imaging–physiology associations, major available demographic and physiological confounders were adjusted where appropriate. Several principal findings remained significant after adjustment, including the LT-related VBM associations in the right cerebellar base, right superior medial frontal region, and right premotor cortex, as well as selected LT-related fALFF associations in the bilateral precuneus. These results provide additional support for the robustness of the main findings, although some weaker associations were attenuated after adjustment.

### 3.4 Group-specific correlations between neural plasticity and endurance performance

After stratification by training level, the uncorrected analyses suggested different patterns of associations across groups. The moderately trained group showed the broadest descriptive distribution of positive associations, including hippocampal fALFF (*r* = 0.684, *p* = 0.042), premotor fALFF (right *r* = 0.889, *p* = 0.001), dorsolateral prefrontal measures (above *r* = 0.77), and cerebellar regions such as the vermis (fALFF *r* = 0.815, *p* = 0.007). These findings suggest that moderate training reflects a highly sensitive phase of neural plasticity related to endurance. These findings may suggest that moderate training is associated with a sensitive phase of neural-physiological coupling, although this interpretation remains exploratory.

High-level athletes demonstrated a more focused neural profile centered on the prefrontal cortex, with the strongest association found between right dorsolateral prefrontal GMV and ILT (*r* = 0.957, *p* = 0.001). This represents the largest effect observed in the dataset and may reflect differences in neural-physiological coupling at higher training levels. This pattern may reflect differences in neural-physiological coupling across training levels rather than definitive differences in neural efficiency.

The control group exhibited a distinct pattern with predominantly negative associations, particularly in the hippocampus and inferior cerebellum, such as the negative association between right hippocampal fALFF and maximal oxygen consumption (*r* = −0.673, *p* = 0.006). This suggests lower neural efficiency in untrained individuals and emphasizes the influence of training level on brain-endurance coupling.

After applying FDR correction, the pattern of surviving associations differed across groups. The moderately trained group showed the broadest distribution of LT_re-related associations, involving parahippocampal, cerebellar, and dorsolateral prefrontal regions. The control group showed a more restricted pattern, with surviving associations primarily in inferior cerebellar and hippocampal regions. The high-level group was characterized by a focused prefrontal profile, with the right dorsolateral prefrontal cortex remaining significant. Nonparametric Spearman analyses generally supported the principal prefrontal effects and control-group findings, whereas several associations in the moderately trained group were less stable after nonparametric testing. Given the modest subgroup sample sizes and partial attenuation of effects in sensitivity analyses, these between-group differences should be interpreted with caution.

## 4 Discussion

### 4.1 Endurance performance is linked to distributed multimodal neural features

Across the full sample, incremental exercise-capacity indicators were associated with structural and functional characteristics in the dorsolateral prefrontal cortex, premotor cortex, precuneus, temporal regions, and posterior cerebellum. These regions are known to participate in motor planning, executive control, interoceptive integration and sensorimotor coordination, which aligns with earlier findings from our group and converging evidence from other studies (Zhang et al., 2025; Zhang et al., 2024). Prior work has shown that endurance athletes tend to exhibit greater cortical thickness or GMV in frontal and motor regions, enhanced cerebellar involvement, and altered default mode or sensorimotor network connectivity (Zhang et al., 2025; Zhang et al., 2024). Research in non-athletic populations has also demonstrated that higher aerobic fitness relates to increased prefrontal activation, stronger parietal engagement and improved cerebellar efficiency, suggesting that endurance capacity depends on widely distributed neural resources rather than a single localized mechanism (Li et al., 2019; Perez-Suarez et al., 2018; Tarumi et al., 2021; Wade et al., 2020; Zhang et al., 2022). The present study extends this literature by demonstrating that multiple physiological indicators of aerobic capacity, including maximal oxygen consumption and lactate-based thresholds, correspond to multimodal MRI features within these networks. This pattern supports the view that graded maximal-effort tolerance is associated with the coordinated involvement of cognitive control systems, motor networks, and cerebellar-cortical integration, while also remaining compatible with broader processes related to effort regulation, interoception, and affective control during strenuous exercise. It should be noted that all physiological measures in the present study were obtained using cycle ergometry. Therefore, these indices primarily reflect tolerance to graded maximal effort and central/metabolic responses, rather than modality-specific performance during incremental exercise. Given the well-established modality specificity of endurance adaptations, particularly in trained runners, the present findings should not be interpreted as reflecting running-specific endurance capacity. Instead, they are more appropriately understood as neural correlates of physiological responses to graded maximal effort under controlled laboratory conditions.

In addition, alternative interpretations should be considered. The distributed neural patterns observed in the present study, spanning prefrontal, parietal, temporal, cerebellar, and subcortical regions, are not specific markers of endurance performance per se, but are widely implicated in domain-general processes, including cognitive control, effort regulation, interoception, and fatigue perception. These regions may support central processes related to cognitive control, motivational regulation, and affective responses during maximal-effort testing. For example, prefrontal and parietal networks have been linked to attentional control and effort allocation, while cerebellar and limbic-related systems may contribute to the perception of fatigue and affective regulation under increasing physiological load.

From this perspective, the present findings are more appropriately interpreted within a brain–effort coupling framework, reflecting integrated central mechanisms that regulate physiological responses during graded maximal-exercise conditions. Accordingly, individual differences in incremental exercise capacity may partly arise from variability in how cognitive, motivational, and affective processes modulate effort during demanding tasks, rather than purely from peripheral or sport-specific endurance mechanisms.

### 4.2 Moderately trained individuals showed broader descriptive association patterns

The moderately trained group showed the broadest descriptive distribution of associations between incremental exercise-capacity indicators and multimodal neural features. This pattern was most evident in prefrontal, premotor, parietal, and cerebellar regions and was broadly consistent with our earlier longitudinal observations. One possible interpretation is that moderate training may coincide with a phase in which brain–physiology coupling appears more widely distributed, potentially reflecting ongoing adaptation across multiple regulatory systems. However, this interpretation should remain tentative given the modest subgroup sample sizes, the partial attenuation of some effects in sensitivity analyses, and the absence of formal interaction testing across groups. Within these constraints, the broader pattern observed in the moderately trained group may still be compatible with mechanistic accounts emphasizing adaptive remodeling under conditions of substantial but not yet highly specialized training load.

### 4.3 High-level athletes showed a more focal prefrontal-associated pattern

High-level endurance athletes demonstrated a focused pattern of associations that were concentrated in the dorsolateral prefrontal cortex. This contrasts with the broader associations observed in moderately trained individuals and suggests a shift from widespread adaptive plasticity toward a more refined and specialized neural profile at advanced stages of training. Such a pattern is consistent with the neural efficiency hypothesis (NEH) (Li and Smith, 2021), which proposes that expert performers engage fewer but more strategically relevant neural resources during both physical and cognitive demands. Within this framework, long-term endurance training may refine prefrontal contributions to effort regulation, performance monitoring and executive control, allowing highly trained athletes to maintain high levels of output with reduced cognitive and metabolic cost. Mechanistically, this concentrated prefrontal involvement may reflect several long-term adaptations. First, repeated exposure to sustained aerobic effort may strengthen synaptic efficiency and network stability in prefrontal circuits that regulate pacing, attention and inhibitory control, functions known to be critical for maintaining performance under fatigue (Da Costa et al., 2017; Thomas et al., 2020), (Zhang et al., 2024), (Zhang et al., 2025). Second, the consolidation of motor and physiological skills over extensive training histories may reduce reliance on broader cortical and subcortical support networks, resulting in a more targeted engagement of prefrontal areas that coordinate cognitive aspects of endurance (Surkar et al., 2025). Third, predictive processing models (Burnston, 2021; Lee et al., 2021) provide another perspective, suggesting that highly trained athletes develop more accurate internal models of effort demands, enabling prefrontal regions to guide motor output and physiological regulation with greater precision and reduced variability.

This interpretation aligns with our previous longitudinal findings, in which high-level athletes exhibited selective increases in prefrontal DC rather than widespread structural or functional change. Together, these converging observations imply that the neural architecture supporting elite performance during incremental exercise becomes increasingly streamlined over time. In this sense, prefrontal regions may serve as a core regulatory hub that integrates interoceptive signals, anticipatory control processes and sustained attentional regulation, supporting the high consistency and stability required for prolonged performance during incremental exercise.

### 4.4 Control participants showed a different descriptive association profile

The predominantly negative associations observed in the control group revealed a neural profile that differed qualitatively from those of the trained groups. These negative relationships, particularly in hippocampal and cerebellar regions, may reflect the greater neural cost required to support physical exertion in individuals with lower endurance capacity. Several mechanistic interpretations offer insight into this pattern. First, less trained individuals may rely more heavily on compensatory activation within memory, emotional regulation and motor coordination regions when physiological demands increase (Avancini et al., 2024; Hyodo et al., 2024). Such compensatory recruitment has been observed in low-fitness or sedentary populations, where greater hippocampal and cerebellar engagement is required to maintain performance on cognitive or motor tasks (Drucker et al., 2019; Paitel and Nielson, 2023; Ziaei et al., 2020). This pattern has been interpreted as reduced neural efficiency, consistent with evidence that individuals with low aerobic fitness often show increased neural effort during both motor execution and executive control tasks. Second, lower fitness levels may amplify interoceptive and affective responses during exercise, leading to heightened engagement of brain regions associated with perceived exertion or emotional distress. Studies in untrained individuals have reported stronger connectivity between limbic structures and sensorimotor areas during physical tasks (Schmitt et al., 2019; Wu et al., 2025), suggesting that fatigue, discomfort and physiological strain impose additional neural burdens. This interpretation is compatible with models that view fatigue not only as a peripheral phenomenon but also as a centrally regulated perception shaped by emotional and cognitive processes. Third, the negative associations observed here may indicate weaker functional integration across motor and cognitive networks in controls. Without the adaptive restructuring that accompanies endurance training, neural systems involved in executive regulation, motor planning and physiological monitoring may operate less cohesively, leading to less favorable coupling between brain metrics and endurance indicators. This contrasts sharply with the coordinated and positive associations seen in trained individuals and reinforces the idea that training history fundamentally alters how the brain supports aerobic performance.

Taken together, these observations suggest that lower training exposure may be associated with a different descriptive pattern of brain–physiology coupling during graded maximal-effort testing, although formal statistical evidence for stage-dependent coupling was not established in the present study.

### 4.5 Implications, limitations and future directions

The present findings offer several implications for the understanding of performance during incremental exercise and its neural foundations. First, the identification of training level dependent neural signatures suggests that endurance capacity is supported by distinct patterns of structural and functional organization that vary across stages of athletic development. These patterns may provide a framework for individualized training strategies, as different stages of adaptation may require different emphases on cognitive control, motor coordination or metabolic conditioning. Second, the convergence of prefrontal, premotor, parietal and cerebellar involvement underscores the importance of integrated central regulation in performance during incremental exercise, complementing traditional physiological models that focus on peripheral systems. This integration may also offer potential neural markers for talent identification and monitoring of training progress.

Several limitations should be acknowledged. First, regarding the testing modality, the use of cycling protocols in a cohort of runners limits our assessment of modality-specific endurance. Second, the sample size within each training group was modest, especially in the high-level group, which limits the statistical stability of our subgroup analyses and constrains the generalizability of the findings. Third, the cross-sectional design precludes causal inferences regarding neural plasticity or temporal dynamics between endurance capacity and brain adaptations. In addition, although descriptively different subgroup patterns were observed across training levels, formal interaction or moderation analyses were not performed. Therefore, the subgroup differences reported here should be interpreted as descriptive and hypothesis-generating rather than as definitive statistical evidence of stage-dependent coupling. Additionally, although multimodal MRI provides complementary insights, the present study lacked concurrent cognitive, autonomic, and affective measures. The absence of these assessments limits a precise mechanistic interpretation of the observed prefrontal and limbic findings, although they may contribute meaningfully to endurance-related neural adaptations.

Future work would benefit from larger cohorts and longitudinal tracking to identify how endurance related neural signatures evolve across training phases. Integrating behavioral and physiological measurements, such as executive function testing, affective evaluations, hormonal profiling and autonomic indicators, may help clarify the mechanisms linking endurance training to neural plasticity. Advanced computational approaches, including multivariate modeling and machine learning, may further support the development of predictive models of performance during incremental exercise based on multimodal neural features. Such approaches could contribute to more precise and individualized training prescriptions and deepen our understanding of the central processes that shape human endurance.

## 5 Conclusion

This study indicates that incremental exercise-capacity measures are associated with coordinated structural and functional characteristics across multiple cortical and subcortical regions. After multiple-comparison correction and supplementary analyses, the surviving findings were more focal than suggested by the uncorrected results. Although descriptively different subgroup patterns were observed across training backgrounds, these findings should be interpreted cautiously as preliminary and hypothesis-generating rather than as definitive evidence of a stage-dependent neural trajectory.

## Data Availability

The datasets presented in this article are not readily available because the dataset contains sensitive human neuroimaging and physiological information that cannot be fully anonymized. Due to privacy protection, ethical approval constraints, and the small sample size that increases the risk of re-identification, the raw MRI and physiological data cannot be made publicly available. De-identified summary data can be provided upon reasonable request to the corresponding author. Requests to access the datasets should be directed to zhangdong94@163.com.
